# State work and the testing concours of citizenship

**DOI:** 10.1111/1468-4446.12743

**Published:** 2020-04-21

**Authors:** Willem Schinkel

**Affiliations:** ^1^ Department of Public Administration and Sociology Erasmus University Rotterdam Rotterdam Netherlands

**Keywords:** borders, citizenship, integration, migration, state, testing

## Abstract

Anyone trying to be a citizen has to pass through a set of practices trying to be a state. This paper investigates some of the ways testing practices calibrate citizens, and in doing so, perform “the state.” The paper focuses on three forms of citizenship testing, which it considers exemplary forms of “state work,” and which all, in various ways, concern “migration.” First, the constitution of a “border crossing,” which requires an identity test configured by deceptibility. Second, the Dutch asylum process, in which “being gay” can, in certain cases, be reason for being granted asylum, but where “being gay” is also the outcome of an examination organized by suspicion. And third, the Dutch measurement of immigrants’ “integration,” which is comprised of a testing process in which such factishes as “being a member of society” and “being modern” surface. Citizenship is analyzed in this paper as accrued and (re)configured along a migration trajectory that takes shape as a *testing concours*, meaning that subjects become citizens along a trajectory of testing practices. In contributing both to work on states and citizenship, and to work on testing, this paper thus puts forward the concept of citizenship testing as state work, where “state work is the term for that kind of labor that most knows itself as comparison, equivalency, and exchange in the social realm” (Harney, 2002, pp. 10–11). Throughout the testing practices discussed here, comparison, equivalency, and exchange figure prominently as the practical achievements of crafting states and citizens.

## 1 INTRODUCTION: STATE WORK AND/AS TESTING PRACTICES

Anyone trying to be a citizen has to pass through a set of practices trying to be a state. Those who become visible as “migrants,” in particular, are subject to a variety of practices aimed at ascertaining their identity, provenance, and formal citizenship status. Such practices often have the character of tests, and this paper discusses three kinds of tests as a way of analyzing the co‐constitutive effects of “states” and “migrants.” These tests can be tests of identity, as when people's passports are checked at the border. They can be tests of veracity, as when the accounts by asylum seekers are scrutinized by state officials. And they can also be tests of belonging, as when social scientific measurement apparatuses are deployed to measure “immigrant integration.” In this paper, I discuss these three cases as ways to shed light on the coproduction of the state and citizenship. I conceive of citizenship as a *testing concours*. That means citizenship is here considered not as an individuated bundle of rights and duties (cf. Marshall, 1963), nor in terms of “acts of citizenship” by subjects themselves (Isin & Nielsen, 2008), nor exclusively as “migrant citizenships” (Nyers, 2015). These are valuable ways of speaking of citizenship, but here I adopt the complementary approach of considering specific trajectories of citizenship as inextricably bound up with state technologies for the visualization of migration.

An increasing literature argues that migration is not an object readily available for observation, but exists by rendering the movement of certain people visible as “migration.” Migration, in other words, does not consist of people travelling from one country to another, but of the registration of the movement of some, but not of others. On the one hand, this perspective builds on work in the “autonomy of migration” literature (Bojadžijev & Karakayali, 2010; Mezzadra, 2010; Moulier‐Boutang, 1998; Papadopoulos & Tsianos, 2013; Rodriguez, 1996), which considers migration as a phenomenon characterized by moments of relative autonomy from either socio‐economic circumstances or state control. On the other hand, it builds on work that emphasizes the visualization of migration. Not only does migration become a spectacle in and through border control (De Genova, 2013), the very “thing” called migration does not become available for public consideration prior to forms of visualization that are constitutive of it. In other words, migration does not precede visualization (Dijstelbloem & Broeders, 2014; Dijstelbloem, van Reekum, & Schinkel, 2017; Scheel, 2013; van Reekum & Schinkel, 2017). What is called “autonomy of migration” might be more suitably termed “autonomy of mobility.” Mobility only becomes “migration” once state officials record data, check passports, grant visa, refuse entry, statistically assess movement, and out of the meshwork of these practices comes the object called “migration,” which remains unstable because it can never be fully traced back to data.

At the same time, visualization, registration, and surveillance should not be seen as emanating from sovereign state bodies with the capacities to visualize. Rather, what this paper discusses as citizenship testing allows an understanding of sovereignty by way of mobility (cf. Papadopoulos, 2018). As Annalisa Pelizza has said, “migrant registration and identification” can be seen as the “co‐construction of individuals and polities” (Pelizza, 2019, p. 1). Seen in this light, migration control and the calibration of people's citizenship enable what Timothy Mitchell has called “state effects” to exist and persist (Mitchell, 1991, 1999). In visualizing migration, there is something to be *decided*, but that is just another way of saying that the specter of sovereignty requires the possibility of performing ritualized operations on the movement of people that cannot be contained, and never were. The visualization and public enactment of what we call “migration,” over against the mere movement of bodies across the earth, thus presents “the state” with ways to prop up itself, to give credibility to the existence of state effects. Sovereignty cannot exist without its ritualized confirmations (Hansen & Stepputat, 2005). To say that migration and the state are co‐constitutive of each other is reductive of the generally asymmetrical ways in which particular practices and associations (the ones we tend to call “state”) attach to other practices and associations (the ones we end up calling “migration”) in order to enact “migration” as, itself, a particular effect of the state. As Mitchell has said, what is called “state” comes out of “methods of organization, arrangement and representation that operate with the social practices they govern, yet create the effect of an enduring structure apparently external to those practices” (Mitchell, 1999, pp. 77–78).

This paper looks at such effects by considering different moments and spaces of “migration” in terms of particular kinds of testing practices. These testing practices constitute the work, which can be properly called “state work” (Harney, 2002), that renders “migration” and “citizenship” not only visible but also more broadly available for practical engagement and for public assessment and concern. State work is to be seen not as the work enacted by the state, but the ongoing process of state formation through particular kinds of labor (i.e., the production of state effects) in which the visualization, calibration, and administration of “migration” and “citizenship” serves as one of several occasions to produce state effects. In the midst of a presumed ongoing “migration crisis” in Europe, this is all the more relevant because, as Nicholas de Genova has said, there is a “plurality of contenders for sovereign power” (De Genova, 2017, p. 5). Likewise, such a perspective contributes to a growing awareness of “de‐naturalizing the national” in migration studies (Amelina & Faist, 2012).

Rogier van Reekum has productively differentiated between the performative effects that pertain to the visualization and calibration of “migration” and what he calls the demonstrative effects that always accompany such performativity but exceed it by drawing attention to the specificity of the association that produces it (van Reekum, 2018). By enacting the public life of “migration,” “the state” demonstrates the contingency and limitations of its own entanglement in the world of territories, materials, signs, and moving bodies. As van Reekum says:

migration is, through its varied and contradictory visualizations, also a test in which the capacities and tendencies of an association—“Europe”, for instance—are demonstrated. (van Reekum 2018, p. 4)

In close proximity to this conception of migration, this paper considers “citizenship” as consisting of a variety of tests. Its main aim is to highlight the different ways in which “the state” is enacted through a state work that takes the form of tests performed on “migrants.” In the same process, the citizenship of the subjects of these tests is enacted in line with an increasing emphasis in Western countries on “earned citizenship” (Van Houdt, Suvarierol, & Schinkel, 2011).

The next section discusses the concept of “citizenship concours.” The following sections discuss three different cases—border crossings, asylum decisions, and immigrant integration monitoring—as sites along a citizenship testing concours. The first of these cases is largely conceptual and theoretical in nature. The second is based on ethnographic data collected by Hertoghs (2019), and analyzed by her and myself (Hertoghs & Schinkel, 2018), and the third is based on a discourse analyses I conducted (cf. Schinkel, 2017).

## 2 CITIZENSHIP AS TESTING CONCOURS

Testing has become central to the configuration of citizenship in Western countries. The spread of police work, as well as of securitization and surveillance, and the multiplication of borders all entail rhythmic setups in which statuses are confirmed or denied, bodies are included or not, and borders are crossed or not. To speak of a “testing concours” in the context of migration and citizenship is meant to evoke the etymological sense of “concours” as “moving, running, or flowing together,” and as concerning both a gathering or accumulation and the tracing of a trajectory. Deriving from *concurrere* (to run together), it at the same time signifies competition and flight. In the context of testing, it recalls notions such as a *concours d’élégance*, a “parade of vehicles in which the entrants are judged according to the elegance of their appearance” (OED, 1923). Something quite close to this happens when physical appearance is compared across face and document at the border (my first case study in this paper). And it occurs when asylum applicants in the Netherlands are tested in terms of their sexual identity (second case). Immigrant integration surveys (third case) likewise have an element of “beauty contest,” of being interpellated to present the self in a morally ideal form that mirrors as closely as possible a benchmarked national norm.

To consider citizenship as a trajectory of tests also means to see it in terms of indetermination, as practices for the calibration of potentiality that, as outcome, steer people in certain directions, enabling or preventing their circulation and their options. Citizenship as test so to speak resides in the impasse between test entry and test result, if only because the outcome of a test tends to give rise to another test. State work here consists of practices and sites of indetermination, of not‐yet, and this waiting mode is indeed typical of common experiences of, and “at” the border. This is true for those who wait to have their passports checked at sites that stand in for “territorial borders” (which most of the time are not “at” the territorial border). But it is also true for those who, in a literal sense, have long since arrived but whose mode of living is nonetheless observed, by means of immigrant integration monitoring, as arrested, as not yet up to speed with modernity, not yet fully “arrived” (Boersma, 2019; Boersma & Schinkel, 2018). This monitoring amounts to a test of the degree to which they have actually “arrived,” the term for which tends to be immigrants’ “integration,” which since 1994 is interchangeable in policy language with “citizenship” in the Netherlands (Schinkel & van Houdt, 2010).

The time of the test, then, condemns one to impasse, to a life on hold. However briefly in some cases (as in the passport check), in others this can be an extended modality of living (as in immigrants monitored in their “catching up” with modernity). Those who reside “in the hold” of such tests experience state work. Their lives are impending, pending the results of tests. But as is so often the case with experiments, tests, or pilots, one outcome of a test is that more tests are needed. Even if one passes a test (one is granted asylum), one is enrolled in yet another series of tests (citizenship or language tests, immigrant integration tests), and so those who emerge from the object of “migration” are not readily cut loose from it, but are rather enrolled in a testing concours that renders their citizenship available for public scrutiny.

A life in the hold of citizenship tests does not mean the total blocking of mobility. Rather, as Tazzioli has shown, it decelerates, obstructs and troubles the trajectories of migrants (Tazzioli, 2017). Precisely through this kind of “migration control,” the state itself emerges as an actor with an address. Because I am interested in the ways testing configures the tester (in this case: “the state”), I will not pay attention here to something that, at first sight, would have been obvious to include: citizenship tests designed by governments to explicitly “test” the degree to which immigrants are “assimilated” to “host societies.” In recent years, much research has appeared on these tests (Bauböck & Joppke, 2010; Etzioni, 2007; Michalowski, 2011; Monforte, Bassel, & Khan, 2019; van Houdt, 2014; van Oers, 2013). In the Netherlands, from which I will draw two of my three cases here, such tests have been subject to critique because of their unrealistic character: hardly any “native Dutch” would pass such a test (who knows what to call the sticks that separate your groceries from the next person at the supermarket?). In the Netherlands, citizenship tests are also conducted prior to immigration, at consulates in “sending countries.” As naturalization tests, they can be quite consequential. Furthermore, as recently shown in the UK, they have real effects in terms of sorting inclusion and exclusion (Monforte et al., 2019). I will not discuss them extensively here for two reasons. The first is that they are part of, or tend to function as, a spectacle, and their attention in both critical and affirmative social scientific work always threatens to contribute to their spectacular mode of operating. The second reason not to include them here is more pertinent as it concerns the work that I would like the concept of a test to do here. This paper proposes thinking about citizenship and migration as trajectories interlaced with, and in part coordinated by, various testing practices that are not necessarily explicitly, deliberately, and consciously planned *as* tests. In particular in the context of this special issue, this is a way of drawing attention to something that tends to be little emphasized: it points at social practices that operate as tests without being explicitly conceived as such. There are practices and situations that function as tests without being imagined as tests. And there are demonstrative effects without demonstrations as the explicit aim. This paper therefore also seeks to push our understanding of social practices as tests, and to do so it is most fruitful to apply the concept of test to cases where that word is not necessarily used.

The following sections contain the three case studies to develop this conception of citizenship as testing concours: (1) identification and border crossing; (2) the establishment of applicants’ sexual identity in the Dutch asylum procedure; (3) the monitoring of immigrant integration. These cases constitute three stops, and three configurations of the hold, in an ongoing concours of citizenship.

## 3 FIRST STOP: CROSSING A BORDER AND THE TESTS OF IDENTIFICATION

In the prevailing vernacular of borders, the figure of the cross is omnipresent. Borders are crossed, and to be able to cross a border is a key feature of citizenship. Movement proceeds orthogonally to territorial lines. Yet an elaborate infrastructure is in place to provide this vernacular with its everyday assumed naturalcy. Infrastructure, by definition, is distributed, and so what gets to be abstractly considered as a line is rendered possible by a concrete but dispersed set of tools and practices. First of all, the line needs stand‐ins in the form of gates, fences, walls, and signs. This spatial semiology cannot be collapsed into a single “line” but it can enact what is understood as the “line” of the border. It also indicates zones where this enactment takes place, and these zones are zones of the hold: people are held up, both in the sense of being slowed down and having to wait “at the border,” and in the sense of potentially being held in rooms or cells, for interrogation or internment. So while on the one hand, the spatial semiology of the border is a gesturing that signals the waning of state power “at the border” (Brown, 2010), on the other hand it enacts zones where that power is displayed in one of its most raw forms. The zone of the hold, however, is a membrane. It exists to permeate, to sift and sort, and therefore it is a zone of testing. Testing results in speeding up again, in the continuation of circulation, or in an extension of the hold. The zone of the hold is structured in a binary way of “go” and “no go.” It is important to note that this account conforms to the semiological gesturing that enacts the border‐as‐line. There are many other ways in which, ex post facto, the border is said to have been crossed, for instance when migrants turn up that have crossed the border “irregularly.” The point here is precisely that the “regular” way of crossing a border requires a testing setup consisting of state work enacting both the border as a line, and the state as a sovereign body.

That binary setup is structured by a seemingly simple test: the match between document and person. Usually the document is a passport, and often it needs to additionally contain a visa or equivalent, which may or may not be a digital registration. The test thus consists of detecting a match between person and document/registration. The determination of a match, or a lack of one, is what is usually called “identification.” Identification is a specific kind of testing procedure in that it involves the correlation between *two separate visual tests* (van Reekum & Schinkel, 2017). Each of these tests involves the attribution of identities to a body that, if identification succeeds, shows up twice and with a sufficiently high degree of resemblance. Identification thus involves two separate series that are correlated so that the same body yields similar results in the two series. For instance, facial attributes of a body are matched to the attributes of a picture in a passport, names pronounced are correlated with names registered, height observed is correlated with officially reported height, and reported place of birth is checked with registered place of birth. The simplest way of stating this, is that a border guard checks certain attributes of a person's passport with certain attributes of the person in front of them. But this is far from a trivial matter and in fact concerns the double construction of, on the one hand, a body assembled from attributes inferred from the passport, and, on the other, a body made up of observed attributes from the person “at” the border.

This conception of identification has two consequences. First, it means that identification is not a process of memory, an assessment of what is already familiar. Rather, it works towards the singular by means of the individuation or instantiation of identities (of race, ethnicity, gender, nationality, etc.) that are translocal—in fact, their translocal character is the very reason the test of “crossing the border” can be performed at all. And, second, it means identification is based on the comparison of one body with a multitude of other bodies, since this is the only way to individuate attributes to a body. Identification, in other words, entails the attribution of the singular by means of comparison from within the plural. It locates the individual within a multitude of comparable individuals that each participate differently in a set of shared attributes. Why does identification involve the correlation of two separate visual tests? The main reason is that it is governed by what Valentin Groebner (2007) has called *deceptibility*. Deceptibility exists in personal identification because identification works by attaching certain markers to bodies that are, of necessity, detachable. I cannot use my body as proof that I am who I say I am, not even in the case of biometrics. Biometrics is merely a way to—potentially—simplify the tests that border work performs. Biometric data from my body need to be matched with their counterpart in the passport. The body cannot stand in for the image of itself. Historically, of course, the possible exception to this is the tradition of the “real” or “authentic image” of Christ (*vera icon*), as in the shroud of Turin, where the image is an index or imprint of the original and as such participates in it. But that tradition, too, is riddled with the paradox of establishing or contesting the authenticity of the authentic image (cf. Belting, 2005, p. 45). The *vera icon* problematic is repeated in the history of photography, in which William Henry Fox Talbot, for instance, considered “nature's hand” to be present (Bredekamp, 2010, p. 186). But while 19th‐century theories of photography often placed themselves in the *vera icon* lineage, later theories contested photography's immediacy (cf. Bate, 2016, pp. 12–13), and with the separation of the photograph from its object, deceptibility re‐entered consideration.

And so identification of necessity involves the matching between two separate series, between bodies and documents. A border guard does not *recognize* a person; she or he *identifies* the person by comparing body and document, and by comparing the body with a multitude of other bodies. Faces look like other faces, which is why they can be identified. But because the passport that goes with the face might be faked, stolen, or otherwise a case of deception, deceptibility governs the border test. For any singular identification, this might be a risk, and border guards are well aware of it. But for identification as such, it is a necessary condition. In order to check whether I am who I say I am, I have been given a document that is separable from my body, and this separability of necessity entails deceptibility, the possibility of my passing without the proper passport.

That deceptibility governs the border crossing as test therefore does not mean the test works towards the elimination of deception. On the contrary, it means that deceptibility remains and is the very condition of the test. Deceptive mimicry, deceit, camouflage, must of necessity be possible precisely because of the comparative character of identification. Persons are compared to other persons making up classes defined by nationality, birth, skin color, and so on. But passports, too, are compared to other passports to test their authenticity. Finally, passports and persons are compared, tested for sufficiency of resemblance in the face of a deceptibility that cannot be dispelled. If bodies, faces, and passports wouldn't look like other bodies, faces, and passports, there would be no possibility of identification. The production of the singular “identity,” and along with it the production of a “go/no go,” is a result of the comparison from within a plurality of bodies, faces, passports. The problem of identification is the problem of linking up bodies and documents, and the necessary separation of the body from the document means that, in each case, that linking up, and thus the individuation of identity, is what is worked towards, not what can be worked with. And the point of identification is to follow through on the consequences of deceptibility, namely the fact that a body can be *in* but not *of* the place it is at.

The go/no go that is the result of this testing procedure is based on the determination where a body belongs, and how it can, accordingly, circulate. But the test here is possible on the basis of a deceptibility it cannot cancel, and that it is not intended to cancel. Testing is possible on the basis of ambiguity, and its aim is a procedurally justified decision. Testing is thoroughly comparative here: it compares both across bodies and between bodies and documents. In order to stabilize such testing setups, state work has required the historical control of the means of deception. This involves commensuration and the establishment of what I have elsewhere called “comparity,” a space of equivalence that enables comparison (Schinkel, 2016). The infrastructure of bodies, buildings, documents, and technologies that the “border” gathers requires comparity spaces, and the historical stabilization of such comparity spaces is another name for “state formation.” The ability to test brings effects into the world, not the least of which is what Timothy Mitchell has termed “state effects”: the effect of an always internal differentiation between “state” and “society” (Mitchell, 1991). The state's ability to establish a “border crossing” by means of a test of identification is possible on the basis of the spreading of carry‐on markers of identity (passports) across a population, so that an individual within that population, when attempting to cross the border, is always already commensurated in the sense that the comparative testing needed for identification can be carried out. This is why one strategy among those seeking to cross borders “irregularly” is to get rid of their passports. Without the required comparability, states have a hard time “processing” persons on the move. The absence of a passport tends therefore to result in longer periods of remaining in the hold of the state, during which other tests are performed to establish people's identity and their deservingness to enter the country—this is what my second case will deal with. For now I would underscore that, because comparative testing “at the border” is what configures the identification of citizens, it also configures their potentialities (go/no go), their capacities to circulate, or their restrictions, their “being on hold” and their being *in* the hold that “the border” constitutes for many today.

Citizenship testing thus begins “at the border,” with the very determination of being able to do so, or of having done so, legitimately. I focus here on the logic of identification but do so at the cost of abstracting what actually happens “at” the border, where persons and bodies are raced and gendered. It is crucial therefore to develop this analysis in line with a growing body of work on migration and intersectionality (cf. Amelina, 2017; Bastia, 2014; Bastia, Piper, & Carrón, 2011). The role of gender and sexuality in migration is part of my second case in this paper. For a next stop along the testing concours of citizenship ensues for many today who flee their country and attempt to cross a border to build up a new existence in a foreign country by applying for asylum.

## 4 SECOND STOP: TESTING DESERVINGNESS IN LGBT ASYLUM APPLICATIONS

For those who apply for asylum, the hold is a very concrete reality. In most, if not all, Western countries, the asylum applicant is interned, placed in a holding facility that is often called “asylum detention.” Ethnographic accounts of asylum detention attest to the experience of lives “on hold,” in extended periods of passive waiting and boredom (Hertoghs, 2019). But here, too, the hold is a time and place of testing. And here as well, the outcome of the test is a decision: either a refugee is rejected (and then either denied the status of “refugee” or referred to another country for assessment), or a refugee is accepted and granted asylum. The test here revolves around the determination of the “deservingness” of asylum. To illustrate how this takes place, I draw on work done by Maja Hertoghs, who studied the Dutch asylum procedure up close in two asylum detention centers between 2014 and 2016 by means of an extended ethnography of the Dutch Immigration and Naturalization Service (IND), involving observations of hearings of asylum applicants, interviews with IND officials and asylum lawyers, observations of meetings between IND officials and document analysis of hearing reports and asylum decisions (Hertoghs, 2019).

Deservingness has a variety of criteria (Bohmer & Shuman, 2008) that concern the establishment of a match between individual attributes and formal (legal and procedural) reasons for granting asylum. The Netherlands is one of several countries where a person's sexual identity can be reason for granting asylum, specifically in the case of lesbian, gay, bisexual and transgender (LGBT) identities. An asylum seeker ascribing to one such identity and claiming to be prosecuted or otherwise in danger because of it in their country of origin, may be eligible for asylum in the Netherlands. However, the IND needs to establish the veracity of both these claims: it needs to ascertain the plausibility of an LGBT identity, and it needs to ascertain the plausibility of this being dangerous for the asylum applicant. The latter might be the easiest part of “the procedure” (the emic name for the asylum procedure). Lists of countries where LGBT persons are in danger are used, so that, for instance, a gay man from Jamaica is almost certain to be granted asylum. The first, establishing sexual identity, is the tricky part. It is also the testing part of the procedure, since that which the state regards as a person's sexual identity—meaning here: the legitimate affordances of that identity in terms of circulation and citizenship—is an *outcome* of the procedure. As the IND instructs its officers: “When an asylum seeker claims he is LGBT, it is up to the asylum seeker to substantiate that claimed homosexuality” (quoted in Hertoghs & Schinkel, 2018). From the commensuration of LGBT to “homosexuality” in just one sentence, one can surmise that much nuance disappears, and yet the IND is adamantly intent on finding the veracity of accounts of sexual identity.

In LGBT cases, the procedure thus becomes a test of one's sexual identity, and this means the asylum applicant is called upon to provide a true and authentic account of their sexual identity. And as Judith Butler has argued, such account‐giving is inherently paradoxical, since it needs to appeal to social norms, from which the terms in which persons give an account of themselves are derived (Butler, 2005, p. 1). The singularity of the self can, therefore, not be expressed other than by abstracting from that singularity, folding it into norms, expectations, and categories in a narrative account that undercuts the singular. We thus encounter a situation similar to the test of identification discussed in the first case: the singular or individuated can only emerge as the outcome of a test that abstracts from the singular and compares with the general, with categories and norms derived from populations, pluralities. And here, too, deceptibility is key in the sense that one's sexual identity cannot be taken at face value, nor can it be trusted as a mere declarative statement. What the IND seeks to establish is precisely the veracity—the plausibility for all practical purposes—of such statements. Deceptibility is very much present in the everyday workings of the procedure in the form of the suspicion that informs everything IND officers do. This suspicion is considered part of their *raison d’être*, as it is the only guarantee of justice in the face of the perceived need to separate the truly “deserving” from the “undeserving” asylum applicants. In the latter case, “state generosity” is considered abused, and so the establishment of the truth of one's sexual identity happens through a skeptical state epistemology.

But how do we go about this? How do we establish a person's sexual identity? Another paradox ensues, because this happens first of all by conceiving of that sexual identity as an innate, deep‐seated personal core or infrastructure of selfhood (Hertoghs & Schinkel, 2018). Yet secondly, it happens by the demand of a credible *performance* of that innate sexual essence. The test of deservingness in the case of LGBT asylum seekers comes down to this, to being able to perform a deep‐seated identity. Of course this performance remains primarily discursive, though embodied comportment is part of it and it is reported on by IND officers working towards a decision on an asylum case. And part of the performance is the ascription to the essentialist conception of sexuality the IND deploys. This results in questions about, and detailed accounts of, moments of realization of one's sexual identity and, where applicable, coming out or getting caught or recognized as being what one is. In a remarkable inversion of the suspicion power tends to display, the IND’s default suspicion is that a person actually belong to the dominant category of “heterosexuality.” The LGBT categories are the non‐normal ones (which is perhaps why they can just as easily become commensurated to “homosexuality”), and to belong in one of them needs to be proven. Even though here, too, ambiguity remains and deceptibility governs the test, an account is to be crafted in such a way that veracity may be argued for in a procedural sense, informing a decision. Because everything hinges on the credibility of performance here, that performance is rehearsed. Lawyers and volunteers from the Refugee Council prep an applicant for the kinds of questions to expect at the hearings, and try to actively shape responses and comportment.

Applicants pass the test of deservingness when their accounts are “credible,” which means they contain enough details, are not frequently “vague” and are coherent overall as elements of an asylum case. While all these are subjective judgments, the IND goes through elaborate efforts to establish the “objectivity” of the decision. This happens through working with different IND officers at different hearings, by having a decision made by officers not present at these hearings, and by allowing for a preliminary decision (“voornemen”) to be challenged by the applicant's lawyer, after which yet another IND officer weighs the evidence. At stake in the test is often an embodied understanding of the meaning of “homosexuality” that conforms to the IND’s highly heterosexual understanding of this. Most enlightening in this respect is a case where asylum was refused and the arguments for this were given in the written decision:

If the applicant were truly homosexual, he would have been able to credibly speak from his inner feelings and observations about what homosexuality contains, certainly now, after he has been in Europe for many years. (quoted in Hertoghs & Schinkel, 2018)

What becomes apparent here is that the test of one's sexual identity (which is at the same time the test of one's deservingness of—partial—citizenship) centers on a conception of sexuality as inner core and infrastructure of personhood. Sexuality is like a mold into which a person's life unfolds. Because this inner core impresses itself on so much of life, both inner and outer, it is a deep structure that nonetheless has surface traceability. So if an account of (in this case) a homosexual identity does not conform to the very conception of sexuality the IND adopts, it may conclude that the identity if feigned. If it were a deep infrastructure of selfhood, it would surface and be coherently traceable. In the case mentioned here, the IND explicates:

His statements involving what homosexuality contains are least of all convincing. In that context he states … that homosexuality means that a man sleeps with a man. Homosexuals do everything that a man normally puts into a woman into a man, they kiss everywhere, and dress up like women. … It is obvious that the applicant presents a very flat and stereotyped image of what homosexuality contains. An image that might be expected of people that mock people with a homosexual nature or who are ignorant of what homosexuality truly is. It is not credible that the applicant, if he really was homosexual, would present such a superficial and shallow image of what homosexuality is. (quoted in Hertoghs & Schinkel, 2018).

What is striking here is that the IND, which itself espouses a markedly binary, heteronormative and hence stereotypical conception of what it calls “homosexuality,” demands of gay applicants a shared non‐stereotypical view of homosexuality. The performance of homosexuality here emerges as the presentation of the right image of homosexuality, which, while binary and heteronormative, must be judged to be too stereotypical for it to pass off as evidence of homosexuality. In this case, that also means the IND officer states that the absence of romantic feelings by the applicant for his sexual partner are considered non‐credible.

For an LGBT asylum applicant, then, the hold presents itself as a prolonged test in which an image of sexual identity gradually surfaces in written reports of hearings, in a preliminary decision and in a final decision of deservingness or undeservingness. This test is riddled with ambiguity, and ultimately it is clear for all participants that the facts cannot decide the issue, just as they cannot do so in scientific tests (cf. Latour, 1987). As in science, the “facts” are the outcome of testing and deliberating among the testers. But unlike in science, what allows the facts to emerge, at least for all practical purposes, is a delegated form of sovereign power that crystallizes, very classically, in what is throughout the procedure called “the decision.” That decision needs to be well founded, and that is why the test exists, and why suspicion—the desire to avoid error—persists throughout the test. But as is the case with all decisions, the decision is ultimately unfounded, and needs to cling to procedural correctness—proper testing conduct—in order to uphold itself in the face of ambiguity and ultimate arbitrariness. This is the case for all asylum requests, not just those based on sexual identity. Any claim to asylum is met with suspicion and refugeeness—and hence deservingness of asylum status—is met with suspicion and put to the test. As in the case of identification at the border, then, the state emerges in, or better yet as, a testing capacity that configures questions of sovereignty prompted by the circulation of bodies as questions of knowledge of the other. And in both cases, ambiguity is not something the state seeks to eradicate. Rather, ambiguity is what allows the state to manifest itself in the first place. As Rogier van Reekum says, if the state were to really eradicate doubt, “it would progressively rid itself of investigative capacities … Government would truly become administration” (van Reekum, 2018, p. 3). Or in other words, the enactment of borders, in these cases, at the same time demonstrates the specific kind of association that makes claims to statehood—that demonstration is the *effect* of state work, but that is a work which can get to be credibly called “state” only *after* the effect.

## 5 THIRD STOP: ASSESSING “IMMIGRANT INTEGRATION”

My last case pertains to what is called, throughout Western Europe, “immigrant integration.” It pertains to those who crossed the border, who have rights, even to many who never migrated, but who are nonetheless observed as different, as not merely “in need of” something called “immigrant integration,” but as simply tested for it, observed *in terms of* “immigrant integration.” Generally, “immigrant integration” is seen in terms of “adjustment to society.” What immediately becomes clear, then, is that immigrants, as well as their children, tend to be regarded as somehow external to the domain of “society” (Schinkel, 2017). Indeed, this is why it is common, for instance in the Netherlands, to speak of immigrants as residing “outside society” or, as in Germany, as making up a “parallel society” (*Parallelgesellschaft*). Of course they are neither in France or Belgium, and if they were, they would not be objects of problematization and integration testing in the Netherlands or Germany (Boersma & Schinkel, 2018). So the fact that they are, somehow, “here” (in the Netherlands) is constitutive of the assumption that they are really not quite here (in “society”). This is why there is regular (longitudinal) state‐sponsored and state‐organized integration monitoring, and it is also why this monitoring is a form of testing. Immigrants and their children *are* “here”—yet what integration monitoring seeks to assess is whether they are really *here* or whether they are, in fact, still in the process of “arriving” (Boersma, 2019; Boersma & Schinkel, 2018). Immigrant integration monitoring closely mirrors the concerns of state policies (Favell, 2003), and this is not very surprising given the fact that often states are the explicit clients, sponsors, and even conductors of the work that integration monitoring constitutes. It relies, moreover, on surveys, population registries, and employment data that are frequently state‐owned and state‐run. In the Netherlands, the Institute for Social Research (SCP) and the Central Bureau of Statistics (CBS) are the main institutions (both are state institutions) who conduct longitudinal integration monitoring surveys and studies, and my analysis here rests on a discourse analysis of work done by these institutions. This analysis proceeds with a Foucaultian emphasis on the production of objects of problematization, and on the stabilization of encompassing onto‐epistemic conceptions of “modernity” and “society,” to the point where such conceptions are naturalized to such an extent that they are unavoidable presuppositions of talk and action precisely under conditions of ongoing endangerment of their naturalcy and stability. The resulting state work can thus be seen as a contemporary modification of the state‐enacted racism Foucault analyzes in *“Il faut defendre la société”* (Foucault, 1997).

Integration monitoring occurs in a variety of ways, centering mostly on “socio‐economic integration” and “socio‐cultural integration.” For brevity's sake, I shall focus on two forms of the latter kind here. The first pertains to the degree of “modernity” that subjects of integration monitoring are tested to display. This happens in various ways, one of which is to consider their “secularity,” which is defined (reductively) as being non‐religious and/or being non‐churchgoing, even though “church” here mostly stands in for “mosque,” and those who might go to church are often not subjects of integration monitoring. The Netherlands is then defined as “secular,” and the fact that about a million people identify as Muslims is not taken as an indication that the country has changed in terms of its religiosity. Rather, the conclusion of this type of measurement is that a million people are at a greater or lesser remove from “Dutch society.” The test that integration monitoring constitutes, operates as a kind of shibboleth here: if a certain feature is present, then one cannot “pass” as an unproblematic “member of society.” The conclusion is that migrants that are more “religious” than “society” is assumed to be (though the latter is not measured in the same monitoring practices) are less “modern,” and hence show a “lag,” as the modernization literature of the 1950s and 1960s often concluded (cf. Lerner, 1958). In a similar way, anthropologist Johannes Fabian (2014) has argued that anthropology has long assumed that difference constituted a temporal lag. Here, the spatial metaphor of “distance from society” is at once coded as temporal lag (as not being with the program of modernity). Such versions of “difference” are the outcome of tests of difference that ratify themselves because they are conducted solely among those who are considered to “differ.”

Something similar occurs in the measurement of “contacts with members of other ethnic groups,” which is a second way of establishing “socio‐cultural integration.” Here, people get lumped into “ethnic groups” by means of pure ascription, and these are compared with “autochthonous Dutch” in terms of the number of contacts members of these groups have with members of other groups. Though officially rescinded by several government research bodies in 2016, the chthonic language of “autochthonous” (literally: from this soil) and “allochthonous” (from other soil) has been pervasive in the Netherlands from at least 1989 (when the Scientific Council for Government Policy started using it) until the present (cf. Geschiere, 2009). The fact that many if not most “allochthones” were and are born on Dutch soil indicates the way these concepts always already operated as strategic markers of problematization. In the analysis of “contacts,” it turns out that “autochthonous Dutch” have the least contacts with members of other groups, that is, white Dutch citizens, relatively speaking, keep to themselves. But this is not considered a test of their socio‐cultural integration or a demonstration of their lack thereof. Rather, what happens is that the tables are turned, quite literally when tables with numbers of contacts are considered: when reporting results, it appears that “contacts with members of other ethnic groups” in fact means “contacts with autochthonous Dutch.” Somehow, those with the least inter‐ethnic contacts are promoted to the rank of unproblematic members of society, the benchmark for others whose contacts with those autochthonous Dutch constitute the test of their “integration.” Of course, it is difficult for members of a variety of ethnic groups to be in contact with autochthonous Dutch, if the latter have little contact with members of other ethnic groups: this is the same phenomenon. So between the introduction of this measurement of socio‐cultural integration and the presentation of its results, this measurement has turned into a test of the degree to which non‐autochthonous Dutch approach autochthonous Dutch, who have become the benchmark for “society.” But this test, which assumes and measures assimilation, can *only* find difference. This is a difference in degrees. One can be more or less different, more or less well integrated, but there will, due to the definition of groups and the elevation of one of them to neutral reference category, of necessity be difference.

Testing here, as often, pertains to an evaluative assessment of how well specific subjects are doing on selected variables. But the evaluation requires a comparative aspect, a benchmark. A comparity space is silently assumed here, and it is called “society” and consists of “autochthonous Dutch” citizens. For non‐autochthonous Dutch, to be a citizen is to approach, as much as possible, a benchmark one cannot possible ever achieve. In other words, the entire setup of integration testing tacitly assumes, from the outset, an image of “society.” This is never defined, though it figures when “secularity” becomes the benchmark for a test of religiosity as a marker for a degree of “integration.” And it figures, in the sense of emerging from the background in a figure/ground reversal, when “contacts with autochthonous Dutch” become coded as the norm for being “integrated” (albeit not for autochthonous Dutch themselves).

Testing can be considered in terms of revealing the unknown qualities of a certain object. At the same time, throughout the history of testing (most significantly psychological and educational testing, but also in much scientific experimentation), the quality involved in the test was considered known in advance. Testing was, in such cases, configured on the basis of its being sensitive to this, but the quantity or degree to which this quality (property, capacity) existed was the unknown and the hoped‐for outcome of the test. In this case, the “quality” amounts to a statement of difference, and testing pertains to the quantity of difference, the degree of “distance from society,” the “degree of integration.” Of necessity, difference, or a “lag,” is the outcome of testing, because testing occurs on the basis of a comparison to a reference category over against which comparative categories are a priori defined as different. In other words, the test produces a “lag,” and necessarily so, as it “benchmarks” on a category to which the tested subjects or categories are a priori *defined* as different. In fact, their prior calibration as different is the very reason for being tested. Testing is thus a highly tautological affair here. It has all the characteristics of a rhetorical device that enables the state to problematize immigrant groups with good reason, since tests governed by “objectivity” show difference.

And so, in a way, one could say that the “real” test resides in whether one is tested at all. Those who are tested will a priori be assessed as beyond the realm of “society” proper, however “well integrated” they are. Those who aren't tested for their individual or group‐level “difference” receive what I have elsewhere called a “dispensation of integration” (Schinkel, 2017).

So while immigrant integration is “measured,” it is indeed more accurate to say that it constitutes a test of the degree of difference of those measured. The real conceit here would be to believe that, given existing conceptions of immigrant integration, something might be learned that wasn't always already internal to the monitoring system itself. Similarly, Niklas Luhmann has argued that the surprise that may follow a scientific test does not present a “realist” argument against the self‐referentiality of scientific knowledge:

The system can gain confirmation by what it already knows: that it operates in an environment … It thereby learns nothing about that environment—except for the fact that it affirms or negates the system’s expectations. The system only ever tests its own expectations. (Luhmann, 1990, p. 261)

In the case of immigrant integration monitoring, at least, such appears the case, since a “self‐projected meaning” underlies the test of immigrant integration from the beginning: “integration” is always already conceptualized as that which supposedly (though this isn't tested) characterizes the ideal of whiteness, of native Dutch with jobs, a nonreligious life characterized by tolerance but also by the exclusivity of their social contacts among themselves.

If integration testing is considered as one enactment of public life through state work, it becomes apparent that the state here attains a strongly moral character. Integration monitoring is a moral monitoring (Schinkel, 2017), and it has the effect of a moral purification of the imaginary domain of “society.” For it appears that, as soon as problems appear, they concern people not fully “integrated in society.” That “society” itself, then, has no problems. If crime is an issue, it is not “society's” issue, but that of individuals external to it, at a remove from it, who are unintegrated and reside “outside society,” even though prisons are generally regarded as “in” society and a vital part of it even. Likewise, “socio‐economic integration” entails the assumption that those at the bottom of what is a socio‐economic hierarchy are really external to the hierarchy. Poverty is not a problem “in society,” but a problem of individuals “outside society,” and they are outside society for the very reason that they are poor, hence insufficiently “integrated.” “Integration” thus becomes applicable in all cases that deviate from prevailing norms, and it thereby becomes a way of morally cleansing the imaginary of “society.” Considering that integration monitoring is a form of state work, it is then important to recognize the moral work that the state performs in order to enact the very imaginary of society.

Testing here configures differentiation vis‐à‐vis a “normal” benchmark, but that means it is relational and necessarily also pertains to the configuration of the benchmark. That is to say that the quality or capacity tested for, and the characteristics of the tester and the infrastructure of testing are not stable objects that precede the test but derive the plausibility of their assumed stability from the test. What “society” is becomes communicable only by way of displaying difference. This requires the a priori inclusion of “society” as a testing benchmark, but that is not how society becomes publicly available as a reference object. Even though the test always and of necessity required a tacit inclusion of “society,” the public availability of this social imaginary emerges as effect *of* the test. In order for that to happen, there has to be a hold. People are imagined as in the hold of their tradition, of religion, of their ethnic minority, their neighborhood, their culture, or their sending country. In short, they are in the hold of their “background,” which starkly contrasts with the “neutrality” and whiteness of “society.” But that imagination itself means that those people, when they are tested for their integration and demonstrated to be in the hold of their “background,” remain in the hold of the very act of coding and imagining. And it means they are in the hold of the state conducting ostensibly epistemic tests upon them.

And this hold can persist into the future along the long lines of migratory affect. There are what one can call “n^th^ generation immigrants” (Schinkel, 2017): those who never actually migrated but descend from “migrants” (but only post‐World War II migrants, not the politically neutralized hominid dispersal of the predecessors of “native citizens”). These continue to produce progeny that constitute a potential testing reservoir, since culture and tradition can be tenacious, and assimilation can take generations. Testing for “immigrant integration,” then, allows what postcolonial literary scholars call “arrival narratives” to be extended (Boersma & Schinkel, 2018; Quayson & Daswani, 2013). Even at this third stop of the citizenship concours, when immigrants are “settling,” they are again stirred into motion in a work of imagination for which testing provides the evidence. Those who migrated, and their children, are still, and potentially perpetually, arriving. State officials measuring integration here show their Derridean feathers: *différer* (to differ) also means *déférer* (to defer to, to postpone). In this sense, a hold persists for subjects of “immigrant integration.” They resemble the man waiting before the law in Kafka's parable *Vor dem Gezetz*, who forever remains outside to be tested, in a trial before even getting to the law, only to find out that the real test consisted in being tested or not. Perhaps the best strategy for citizenship when confronted with immigrant integration surveys would be what Kafka (1996, p. 380) writes in another one of his short stories, *Die Prüfung* (“The Test”): “He who does not answer the questions has passed the test.”

## 6 CONCLUSION: ON TESTING

By discussing these cases, this paper seeks to primarily contribute to work on the entanglement between the state and migration. A secondary contribution it makes is to understandings of testing in political settings. In this concluding section, I in particular draw out some points regarding the relationship between the state and testing.

Seeing citizenship testing as state work means considering it as “state labor under capitalist conditions” where, as Stefano Harney has said, “state work is the term for that kind of labor that most knows itself as comparison, equivalency, and exchange in the social realm” (Harney, 2002, pp. 10–11). Throughout the testing practices discussed here, comparison, equivalency, and exchange figure prominently. Testing thus involves comparity work, the work of crafting a *tertium comparationis*, a shared space of comparison (Schinkel, 2016). This means that the tests are always already inscribed in a calibrated conception of, in this case, a national norm. Especially in the last two cases it becomes very apparent that testing occurs within an overall orientation toward norms of heteronormativity, nativity, and whiteness.

This “always already” is possible because the practices described here are forms of state work. Testing, like experimentation, involves attention and experience. It's a very pragmatic thing, and if state work amounts to testing in the cases discussed, that means “the state” gets to be seen in a pragmatist conception as a practical achievement. But even here, something of what Cassirer (1946) called the “myth” of the state remains. In their history of wonders, Daston and Park argue that natural wonders, secrets and experiments are all phenomena of experience, and all were “proven recipes for medical and magical preparations, [which] often drew on the occult properties of natural substances, and they were excluded from natural philosophy for the same reasons” (Daston & Park, 1998, p. 129; cf. Hadot, 2004, pp. 165–166). And upon closer scrutiny, the practices that constitute “migration” as a testing concours all have their “occult properties.” They are strikingly characterized by imaginaries of "objectivity,” and they appear as knowledge practices. But all the while, they are also ways of proceduralizing what necessarily escapes: they identify in the face of deception and ambiguity, they establish sexual identity as if it were a deep‐seated infrastructure of being, and they mobilize a mythic “society” in its absence by rendering difference available for attention and further problematization. The kinds of state work discussed here keep a variety of factishes afloat, such as a “society” with “members,” people that “are” (or are not) “modern.” And these factishes, in turn, are assumed as the epistemic foothold for tests of the veracity of identity and citizenship of those enmeshed in the thing called “migration.” They become, in other words, ways of processing variety.

Testing is an adequate practice for processing variety, because it involves differentiation. The necessity of differentiation is the working assumption of all testing; entities with necessarily homogeneous qualities and quantities need not be tested, they can be classified. Because testing involves differentiation it is a practice that figures in the workings of what Deleuze and Guattari (2004) call the “binary machines” that produce the lines of “hard segmentarity” that run through social life: gender, sex, race, class, citizenship, and so on. And these are not mere classifications. As this paper has sought to illustrate, a variety of epistemic practices exist to construe such segmentary lines as “outcomes” of tests. And because these tests are conducted in and through state work, their outcomes are not evenly challengeable. They depend on decisions, that is, on the authority to, as Marylin Strathern has said, “cut the network” (Strathern, 1996). Considering citizenship as a testing concours means to thus situate and relocate what is generally called “state authority.”

Key to the relationship between the state and citizenship testing is that the configuration of tests entails the co‐configuration of the tester. This also means that tests need not be, as Hanson (1993, pp. 18–19) holds, “representational techniques” in which the information collected is not the goal but a representation of it (as an IQ test, for instance, tests not the ability to answer questions but a more general thing named “intelligence”). Yet considering citizenship as testing concours illustrates how “migration” and “citizenship” are enacted through a state work that co‐configures a social “inside” that is presumed as the pre‐existing ground of the test, whether it goes by the name of “nation” or of “society.” This is because the citizenship testing has *public* effects: they both render citizenship and migration available as objects of concern to publics, and they render certain publics available to observation by themselves and others.

One potential limitation of a focus on testing is that one tends to consider setups and practices as consciously designed tests. Thereby, a cognitivist bias is smuggled into the analysis of testing practices. And then “testing” threatens to become a form of deliberation by other means—something that is especially consequential when testing and experimentation are proffered as alternatives (one might say functional equivalents) of liberal, deliberative models of politics. This may be due to the legacy of STS and science studies, and their transposition of the vocabulary and practice of the laboratory (cf. Latour, 1988). In science, tests, experiments, demonstrations, and pilots yield surprise (i.e., information), repetition (redundancy), and ambiguity, but they only ever do so in setups controlled and consciously planned as tests. But the productivity of concepts like test, demonstration, and experiment might be maximized if using them can yield fresh perspectives on phenomena not consciously shaped as tests, demonstrations, or experiments, phenomena that are neither evidence of a “test drive” or part of a rhetoric of testing (Ronell, 2005). A final contribution this paper seeks to make to work on testing lies, then, in considering practices as tests that are not necessarily imagined as such by those involved in them. Paying attention to citizenship and migration in terms of testing reveals that testing involves boundary work, normative benchmarks, and factishes that all concern the configuration of the “test”—in this case: the state and the idealized fiction of the “native citizen” it deploys but cannot substantiate (and thus govern) without testing others.

This also draws attention to an ethics of testing. The state work through which citizenship is configured as a testing concours draws people into policing relations to specific bodies exhibiting specific kinds of mobilities. To presume to be able to test, to assume the power to demonstrate, may be disguised in the objectivity of a modest witness, but it enacts sovereign power and configures the relational as a series of asymmetric capacities to extract exposition effects from bodies. In the cases described here, these asymmetric capacities and the normative benchmarking this comes with, shape a particular ethics of testing in which relationality gets done in asymmetric, suspicious, decisionist ways. Yet, as we know, there are other modalities of relationality. To shape relationality as being‐in‐common, another meaning of “testing” might be more pertinent: testing as displacing or suspending boundaries, as the open and demonstrative commoning of a form‐of‐life that does not pretend to already know how to live together.

## Data Availability

The ethnographic data that support the findings of this study are not publicly available due to privacy or ethical restrictions. The data on immigrant integration surveys are in the public domain.
